# Transient Resolution of Foveal Schisis Following Macula-Involving Retinal Detachment in Juvenile X-Linked Retinoschisis

**DOI:** 10.1155/crop/3486067

**Published:** 2025-09-29

**Authors:** Mohammad Zarei, Nazanin Ebrahimiadib, Parichehr Ghahari, Zohre Ebrahimi

**Affiliations:** ^1^Farabi Eye Hospital, Tehran University of Medical Sciences, Tehran, Iran; ^2^Ophthalmology Department, College of Medicine, University of Florida, Gainesville, Florida, USA

## Abstract

We present the case of a 9-year-old boy with X-linked retinoschisis (XLRS) who experienced transient resolution of foveal schisis after developing macula-involving retinal detachment. Following successful scleral buckling surgery and retinal reattachment, foveal schisis reappeared.

## 1. Background

Juvenile X-linked retinoschisis (XLRS) is the most prevalent form of hereditary macular degeneration in males. It is attributed to mutations in the *RS1* gene, which encodes the protein retinoschisin.

Retinoschisin, mainly expressed in bipolar and photoreceptor cells, is crucial for normal adhesion, interaction, and structure of the retina [1]. A recent study of 52 patients with XLRS found macular retinoschisis in nearly all (99.0%, 103/104) eyes; however, only 76% showed retinoschisis within the central 6 mm, 23% in the central 3 mm, and solely 1.2% in the central 1-mm zone. Peripheral retinoschisis was detected in 46.2% of eyes, and XLRS-related retinal detachment (RD) developed in 7.7% (8/104) of cases [2]. Here, we present a case of XLRS complicated by RD accompanied by resolution of foveal schisis. However, foveal schisis rapidly recurred following retinal reattachment with scleral buckling (SB).

## 2. Case Report

**Figure 1:**
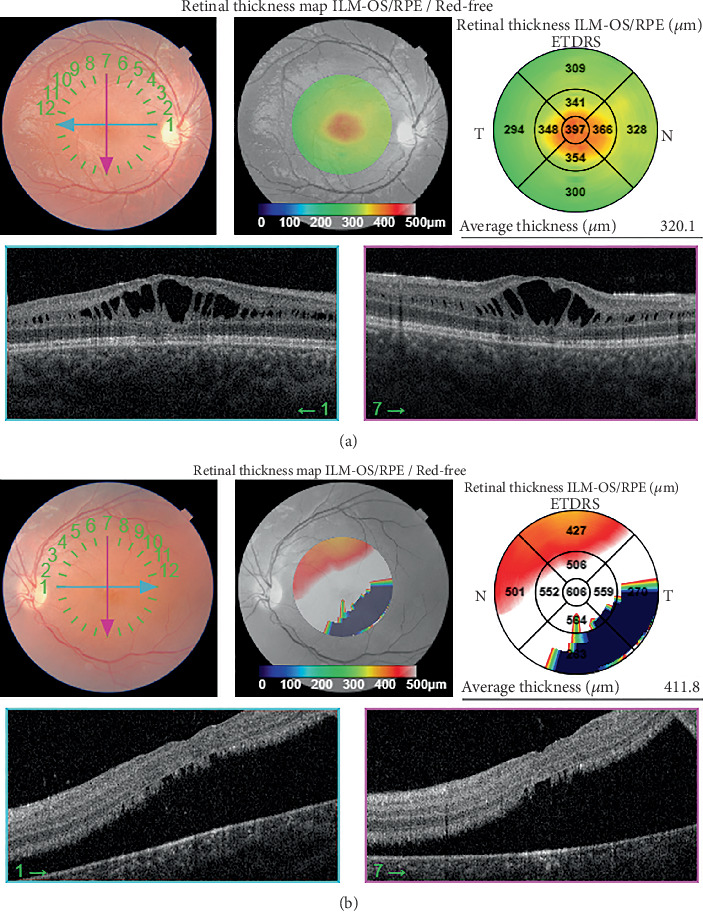
Macular OCT demonstrating foveal schisis in the right eye (a) and macular detachment without foveal schisis in the left eye (b).

A 9-year-old boy with a diagnosis of XLRS was referred for vision reduction in his left eye for 1 month. Best-corrected visual acuity (BCVA) was 3/10 in the right eye and counting fingers (CFs) at 50 cm in the left eye. Anterior segment examination was unremarkable. Intraocular pressure (IOP) with Goldmann applanation tonometry was 10 mmHg in the right eye and 6 mmHg in the left eye. Ophthalmoscopy revealed foveal and peripheral retinoschisis in the right eye and peripheral retinoschisis, macular detachment, vitreous veils, and large inner and outer retinal holes in the left eye. Macular spectral-domain optical coherence tomography (SD-OCT) of the right eye revealed foveal retinoschisis cavities with splitting in the outer plexiform layer, disruption of the ellipsoid zone, and an increase in central foveal thickness (Figure 1a). Macular SD-OCT of the left eye confirmed RD without foveal retinoschisis (Figure 1b).

**Figure 2:**
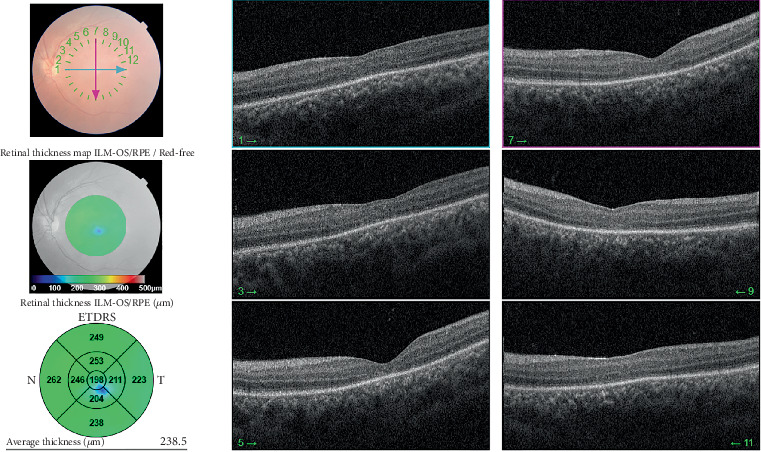
Left eye macular OCT 1 week after scleral buckling surgery shows retinal reattachment and the absence of foveal schisis.

**Figure 3:**
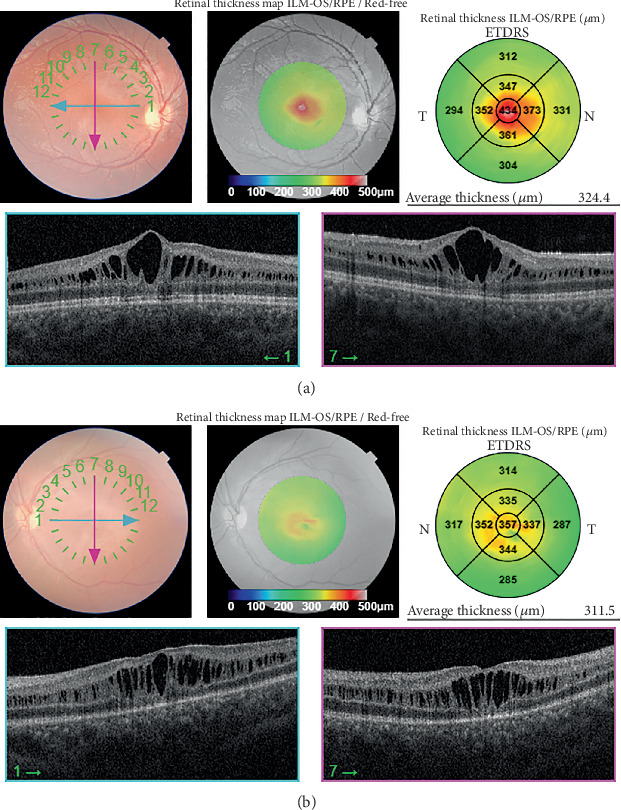
Macular OCT of the right (a) and left eyes (b) 1 month after scleral buckling. Formation of foveal schisis cavities is evident.

Patient underwent SB surgery with a 360-degree encircling silicone band 240 and tire 276 and subretinal fluid drainage. Then, 1 week after surgery, BCVA improved to 1/10, and SD-OCT confirmed an attached retina with no foveal schisis (Figure 2). Then, 1 month after surgery, the patient's visual acuity was 2/10, and SD-OCT revealed the reappearance of foveal schisis in the left eye involving the inner nuclear and outer plexiform layer (Figure 3b). Following the recurrence of foveal schisis, topical acetazolamide (one drop in both eyes every 8 h) was initiated, and despite continued treatment for 1.5 years, the foveal schisis persisted without improvement.

## 3. Discussion

Here, we reported a case of XLRS with the absence of foveal schisis in the detached retina and the emergence of foveal schisis 1 month after retinal reattachment. Below, we propose several explanations for the disappearance of foveal schisis in the detached retina.

First, it is plausible that in detached retina, defective retinoschisin is less produced. The accumulation of abnormal retinoschisin, a cell adhesion molecule in and around Müller cells, leads to the formation of foveal schisis cavities in XLRS [1, 3]. Spontaneous collapse of these cavities has been reported in some older patients [4], which is attributed to decreased production of defective retinoschisin with aging. Retinoschisin is predominantly secreted by photoreceptors, and it is reasonable to presume that with RD, a reduction in its production may occur that will be resumed following retinal reattachment.

The second theory is the relieved vitreoretinal traction in detached retina. RD is reported in nearly 7.7% of XLRS patients. Surgery is recommended for RD or vitreous hemorrhage in XLRS [2]. Previous studies on XLRS patients who were treated with vitrectomy have suggested a role for the vitreoretinal interface in the formation of foveal schisis [5]. One proposed explanation for the disappearance of foveal cysts in older patients is the occurrence of posterior vitreous detachment. Accepting the proposed role of vitreoretinal traction in foveal schisis, macula-involving RD can theoretically reduce the vitreomacular traction and lead to the resolution of foveal schisis. Following the foveal reattachment and re-establishment of traction, foveal schisis can reinstate. However, using vitrectomy to repair RD, tractions are eliminated, and schisis should not recur. Therefore, reports of schisis reappearance in this setting disprove the role of vitreomacular traction in foveal schisis [6]. In this context, some authors emphasize the potential role of posterior tractional forces involving the retinal pigment epithelium (RPE) and choroid [5–9]. SB can remodel the forces between RPE and choroid; however, schisis cavities developed in the fovea in our case.

The third hypothesis is that RD allows the drainage of intraretinal fluid either through inner retina holes to the vitreous or through the outer retinal holes to the subretinal space. With retinal reattachment and closure of holes, drainage is stopped, and schisis cavities reappear.

In our case, the complete absence of foveal schisis in detached retina and relatively rapid development of foveal schisis following reattachment with SB is remarkable. The capacity of the proposed theories remains to be elucidated.

## 4. Conclusion

in XLRS, RD can result in the resolution of the foveal schisis. With retinal reattachment following SB, foveal schisis reappeared. Although the underlying mechanism is not fully understood, decreased retinoschisin, changes in the resultant tractional forces, and/or drainage of intraretinal fluid into the vitreous or subretinal space may have role(s) in this observation.

## Data Availability

The data that support the findings of this study are available on request from the corresponding author. The data are not publicly available due to privacy or ethical restrictions.
